# Neurobehavioral Differences of Valproate and Risperidone on MK-801 Inducing Acute Hyperlocomotion in Mice

**DOI:** 10.1155/2022/1048463

**Published:** 2022-02-23

**Authors:** Po-An Chen, Hui-Yi Wang, Chien-Lun Sun, Mao-Liang Chen, Yi-Chyan Chen

**Affiliations:** ^1^Department of Psychiatry, Taipei Tzu Chi Hospital, Buddhist Tzu Chi Medical Foundation, New Taipei City, Taiwan; ^2^Department of Research, Taipei Tzu Chi Hospital, Buddhist Tzu Chi Medical Foundation, New Taipei City, Taiwan; ^3^Department of Psychiatry, School of Medicine, Tzu Chi University, Hualien, Taiwan

## Abstract

**Objective:**

The glutamate system plays a major role in the development of neuropsychiatric disorders such as addiction, epilepsy, dementia, and psychosis. MK-801 (dizocilpine), an uncompetitive *N*-methyl-D-aspartate (NMDA) receptor antagonist, could increase locomotor activity and stereotyped neurobehaviors mimicking schizophrenic-like features in the mouse model. The study would explore the neuropharmacological differences of risperidone and valproic acid on the MK-801-induced neurobehavioral changes.

**Methods:**

The subjects were male C57BL/6J mice obtained from the National Laboratory Animal Center. Drug effects were assessed using the open field with a video-tracking system and gaiting tests. After habitation, risperidone (0, 0.1 mg/kg) or valproic acid (0, 200 mg/kg) was injected and ran locomotion for 30 mins. Sequentially, mice were followed by intraperitoneal injection (i.p.) with MK-801 (0, 0.2 mg/kg) and ran locomotion for 60 mins. Gaiting behaviors such as step angles, stride lengths, and stance widths were measured following the study drugs.

**Results:**

The results showed that risperidone and valproic acid alone could not alter the locomotor activities. Following the MK-801 injection, the travelled distance and speed in the entire open field dramatically increased. The dose 0.1 mg/kg of risperidone could totally inhibit the MK-801-induced hyperlocomotion compared with that of the saline-injected group (*p* < 0.001). The valproic acid (200 mg/kg) partially suppressed the hyperlocomotion which is induced by MK801.

**Conclusion:**

The more dominant effect of risperidone to rescue MK-801 induced hyperlocomotion compared with that of valproic acid. The partial suppression of valproic acid may imply the psychopharmacological evidence as adjuvant effect to treat psychotic patients through tuning glutamatergic neurotransmission.

## 1. Introduction

Under the clinical circumstance, the agitation, aggression, and psychosis are devasting symptoms in major psychiatric disorders including schizophrenia, bipolar affective disorder, substance intoxication, dementia, and epilepsy [1]. Such disruptive symptoms can significantly raise the healthcare burden including the emergency department, hospitalization, and outpatient setting and even in the community [2]. Although there are numerous neuroscience researches to explore the basic mechanisms of psychotic disorders, to date, there is a lack of convincing reports in the understanding of neurobiological mechanisms underlying psychosis. Growing evidence demonstrates that the glutamate system plays a major role in the development of addiction, schizophrenia, dementia, and other mental disorders [3–6].

Risperidone, an atypical antipsychotic, is widely used to control the acute psychosis through reducing hyperdopaminergic neurotransmission. It is not only approved to treat schizophrenic disorder but also considered as a mood stabilizer in various bipolar treatment guidelines including the World Federation of Societies of Biological Psychiatry (WFSBP) [7, 8], National Institute for Health and Care Excellence (NICE) [9], and Canadian Network for Mood and Anxiety Treatments (CANMAT) [10]. Valproic acid, an anticonvulsant, is used as a mood stabilizer and is proved in the above treatment guidelines for bipolar affective disorders. Treatment guidelines also highlighted that the augmentations of valproic acid and atypical antipsychotics, such as risperidone, have favorable treatment effect over bipolar patients who failed to respond to monotherapy.

In the past two decades, the interactions between psychosis and the glutamate system have been proposed by the *N*-methyl-D-aspartate (NMDA) receptor hypofunction, as the administration of NMDA antagonists has been observed to contribute to psychotomimetic actions other than sedation, anesthetic, and antidepressant effects [11, 12]. Abusers of NMDA receptor antagonists, such as ketamine and phencyclidine (PCP), would experience agitation, perceptual distortions, hallucinations, and delusions resembling positive symptoms of schizophrenia [4, 6, 13]. The addictive properties of these psychoactive substances are related to their tuning the dopaminergic and glutamatergic neurotransmission in certain brain areas such as the prefrontal cortex, ventral tegmental area, and nucleus accumbens, deriving from the NMDA receptor blockade [14]. It will be a curious issue to find a treatment module attenuating the agitated and psychotic symptoms in the subjects with psychoactive addiction.

MK-801 (dizocipline), an uncompetitive NMDA receptor antagonist, has been proven to induce acute excitation, agitation, or aggressive behaviors in different animal models [5, 15, 16]. Memory impairment and learning impairment were also seen in the MK-801 animal model, resembling negative symptoms of schizophrenia through modulation of Rho family protein-related signaling [17]. MK-801-induced neurobehavioral alteration was adapted as an acute schizophrenic-like model in animal studies [15, 18]. Since antipsychotics act to reduce the hyperdopaminergic neurotransmission, the interactions between dopamine and glutamate neuron may play an important role in the development of psychosis from the treatment modality. In animal model, the MK-801-induced hyperlocomotion could be reduced by antipsychotic administration such as risperidone [19] and the results were compatible to clinical findings of risperidone treatment of active psychotic patients. There was a report that valproic acid could prevent the induction of MK-801 sensitization and block the behavioral cross-sensitization of methamphetamine and MK-801 [20]. Clinical evidences have showed the effectiveness of adjunctive therapy by valproic acid and antipsychotics for treating schizophrenia with symptoms of agitation, aggression, and excitation [21–25]. Valproic acid has also been adapted in treatment guidelines for the treatment of aggression and hostility symptoms in schizophrenia [26]. However, the neurobiological mechanism of the valproic acid effect on psychosis remains unknown [27]. Currently, the psychiatric diagnostic systems could not deduce the agitation and disorganized behaviors into one singular diagnosis. Such behavioral manifestations could be a part of diverse psychiatric disorders, including schizophrenia, affective disorders, substance intoxication, and withdrawal state [28–30].

Until now, there is a lack of neurobiological basis to support the pharmacodynamic effect of valproic acid to control the agitated/aggressive features in psychotic patients. To test the valproic acid for potential interactions with glutamatergic and dopaminergic neurotransmission, the MK-801-induced hyperlocomotion was used as a positive control and compared to the risperidone pharmacological reaction. The valproic acid and risperidone were administrated alongside or in combination with MK-801. The study measured the neurobehavioral changes following the separated drug injection and assessed the suppression effect on MK-801. In addition, we also compared the differences of locomotor behaviors and gaiting parameters following valproic acid and risperidone treatment.

## 2. Methods

### 2.1. Animals and Drug Preparations

Subjects were adult male C57BL/6J mice obtained from the National Laboratory Animal Center. Mice were housed as four in a cage in a temperature (22 ± 1°C) and humidity (50 ± 5%) well-controlled vivarium, under a 12-hour light/dark cycle with ad libitum access to food and water. MK-801 and valproic acid sodium salt (Sigma-Aldrich Co.) were dissolved in saline. The risperidone (Sigma-Aldrich Co.) was dissolved in 2% dimethyl sulfoxide (DMSO) and diluted in saline. The drugs were diluted for the subjects to receive 0.2 mg/kg of MK-801, 200 mg/kg of valproic acid, and 0.1 mg/kg of risperidone as injection volume by 10 mL/kg. All experimental procedures were approved by the Institutional Animal Care and Use Committee, Buddhist Taipei General Hospital, and were conducted following the regulation of reduction and refinement. All efforts were made to minimize the number of animals used and their suffering.

### 2.2. Open Field Test

After adaptation to laboratory and housing conditions for 60 minutes, subjects were placed in the open field for a 30-minute habituation. The open field apparatus was a square-shaped opaque device with a length and width of 40.5 cm and 35 cm in height made with acrylic material. It featured a square-shaped central region measuring 28 cm in length and width with central luminous being set at 50 ± 5 LUX. After the habituation period, subjects were randomly divided into three groups, receiving risperidone 0.1 mg/kg, valproic acid 200 mg/kg, and vehicle, through intraperitoneal injection (i.p.). The mice were immediately placed in the open field for locomotion measurement for 30 minutes. Sequentially, MK-801 (0.2 mg/kg) or saline was injected and mice were placed in the open field testing for 60 minutes; the experimental design is demonstrated in Supplement 1. The locomotor activities were recorded via a video-tracking system (SINGA, TW), as shown in Supplement 2 (marked as blue lines). The general experiment procedures were well conducted by negative and positive controls, saline and MK-801, respectively. The drug testing study was separately performed and combined to compare the difference in the same module.

### 2.3. Gaiting Test

The gaiting test was conducted to evaluate the behavioral quality of the mouse behavior. [31]. The gait recording apparatus was a rectangle-shaped device measuring 5 cm in width and 80 cm in length. The luminous was set at 120 ± 10 LUX from the starting point of the apparatus, and 70 ± 10 LUX at the end of the apparatus. The environmental luminous was set at 15 ± 5 LUX. After adaptation to laboratory and housing conditions, subjects were randomly divided into three groups, receiving risperidone 0.1 mg/kg, valproic acid 200 mg/kg, and vehicle, through intraperitoneal injection (i.p.). Subjects were then wetted by ink on hind paws and placed on the gait recording apparatus with strip of papers to record the gaits as the subjects proceed from the starting point to the end. After the first injection, gaits were recorded at 0, 10, 20, and 30 minutes postinjection. Sequentially, mice were followed by a second intraperitoneal injection with MK-801 (0.2 mg/kg) or saline and gaits were assessed at 0, 10, 20, 30, 40, 50, and 60 minutes postinjection. Parameters of the gait were then measured and recorded including the step angles, stride lengths, and stance widths. In the gaiting test, the three consecutive longest footprints were used to measure each parameter. Step angles were measured by the connected lines of the outer border of the hind paws. Stride lengths were measured by the distance between the front edge of the hind paws. Stance widths were measured by the vertical distance between one outer edge of the hind paw and the connected line of the stride length on the opposite side. The mean value was used to represent each parameter.

### 2.4. Statistical Analysis

All the statistical analyses were conducted by GraphPad Prism (GraphPad Software Inc., San Diego, CA) version 5.01.336 and Statistical Package for Social Science (SPSS Inc., Chicago, Illinois). Paired *t*-test and analysis of variance (ANOVA) were performed to analyze the differences of measurement parameters of the mice locomotor activities. Post hoc analysis by Scheffe's multiple comparison test was used in this study.

## 3. Results

### 3.1. The MK-801 Effect

**Figure 1:**
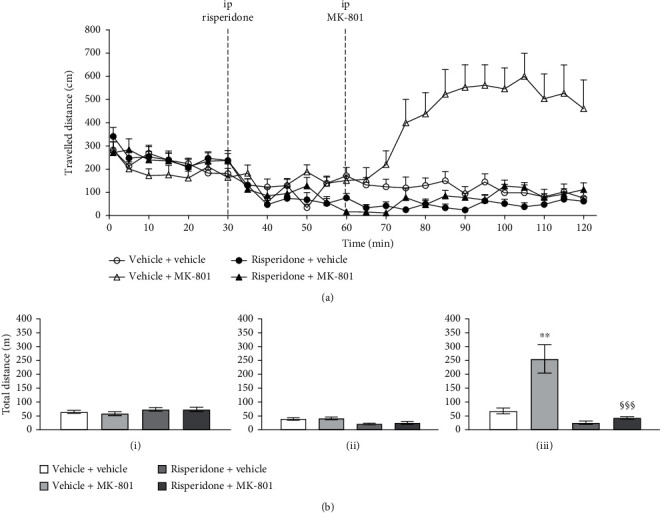
The travelled distance (cm) at the corresponding time points in the open field test following the risperidone and MK-801 injection (a). Comparisons of the total travelled distance (m) for each drug treatment group (b); vertical bars represent SEMs: (i) represents baseline within 0–30 minutes, (ii) represents risperidone drug effect during 30–60 minutes, and (iii) represents MK-801-injected travelling change during 60–120 minutes; total travelled distances are shown in meters. Subject numbers were 9–10 in each group. Differences among the study groups were evaluated by ANOVA and post hoc analysis. Statistically significant differences between groups: ^∗^*p* < 0.05, ^∗∗^*p* < 0.01, and ^∗∗∗^*p* < 0.001 vs. vehicle + vehicle; ^§^*p* < 0.05, ^§§^*p* < 0.01, and ^§§§^*p* < 0.001 vs. vehicle + MK-801.

**Figure 2:**
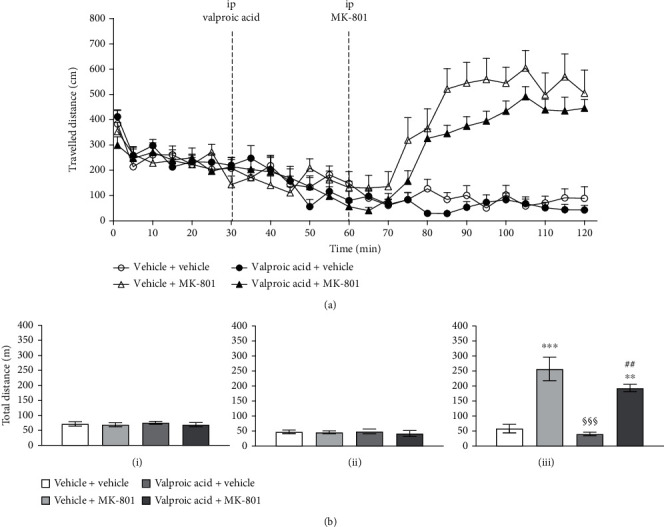
The travelled distance (cm) at the corresponding time points in the open field test following the valproic acid and MK-801 injection (a). Comparisons of the total travelled distance (m) for each drug treatment group (b); vertical bars represent SEMs: (i) represents baseline within 0–30 minutes, (ii) represents valproic acid drug effect during 30–60 minutes, and (iii) represents MK-801-injected travelling change during 60–120 minutes; total travelled distances are shown in meters. Subject numbers were 10–11 in each group. Differences among the study groups were evaluated by ANOVA and post hoc analysis. Statistically significant differences between groups: ^∗^*p* < 0.05, ^∗∗^*p* < 0.01, and ^∗∗∗^*p* < 0.001 vs. vehicle + vehicle; ^#^*p* < 0.05, ^##^*p* < 0.01, and ^###^*p* < 0.001 vs. valproic acid + vehicle; ^§^*p* < 0.05, ^§§^*p* < 0.01, and ^§§§^*p* < 0.001 vs. vehicle + MK-801.

**Figure 3:**
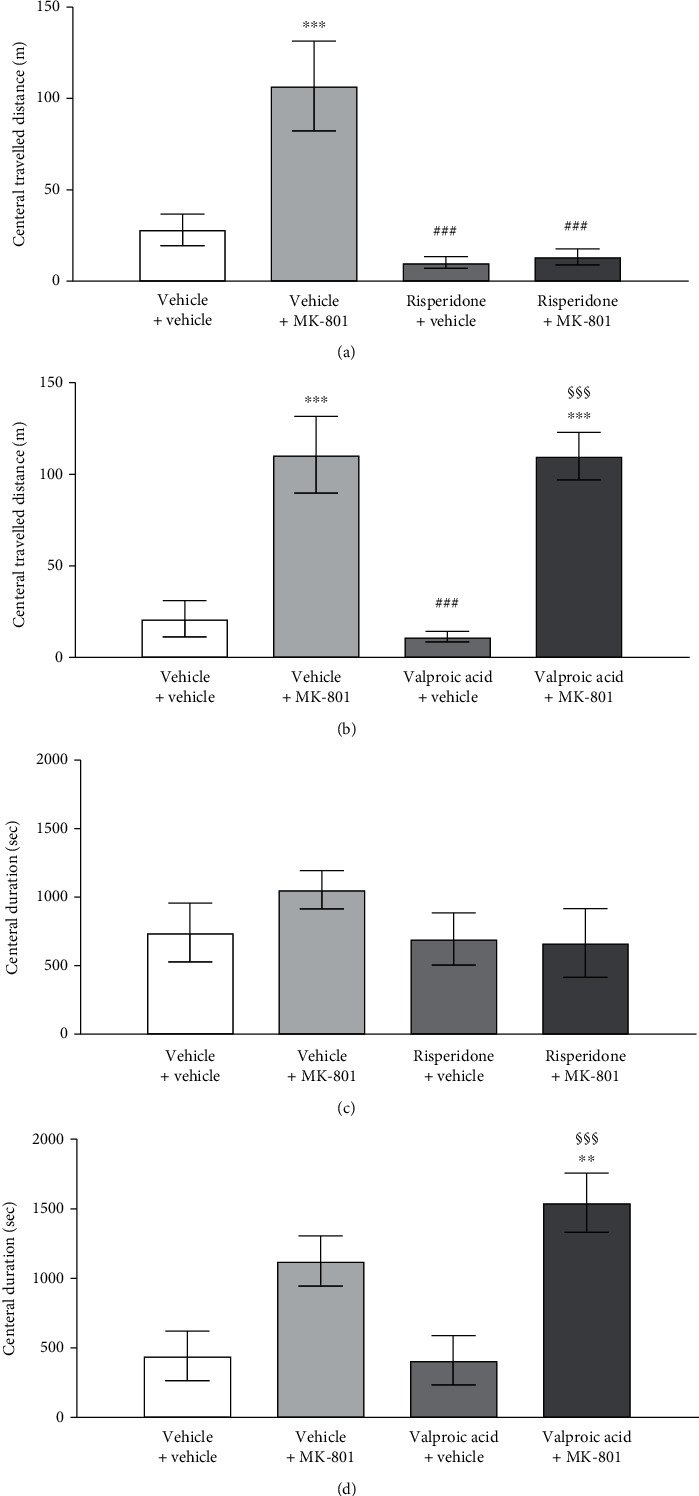
Alterations of the central travelled distance (a, b) and central duration (c, d) followed by MK-801 injection in the open field for mice treated with risperidone/valproic acid. Central travelled distances and duration are shown in meters and seconds, respectively. Vertical bars (for clarity, only the upper or lower portions shown) represent standard errors of the means. Statistically significant differences between groups were evaluated by ANOVA. ^∗^*p* < 0.05, ^∗∗^*p* < 0.01, and ^∗∗∗^*p* < 0.001 vs. vehicle + vehicle; ^#^*p* < 0.05, ^##^*p* < 0.01, and ^###^*p* < 0.001 comparing with vehicle + MK-801; ^§^*p* < 0.05, ^§§^*p* < 0.01, and ^§§§^*p* < 0.001 vs. valproic acid + vehicle.

In the open field test, there were no significant differences in the travelled distance, travelled speed, frequency of entering central area, and duration in the central area between all groups in the habituation period. The mice displayed avoidant behaviors of travelling to the central area compatible with the lower percentage of central duration and central travelled distance. In the open field test, after MK-801 injection, the vehicle + MK-801 group showed a dramatical increase in the total travelled distance and travelled speed in the entire open field. The central travelled speed, frequency of entering the central area, and central travelled distance were significantly increased (*p* < 0.001). (shown in Figures 1–3).

### 3.2. The Risperidone/Valproic Acid Effect

The valproic acid or risperidone alone did not affect the travelled distance. Following the 1^st^ i.p. injection (risperidone or valproic acid), there was no significant difference in the travelled speed, frequency of entering the central area, and duration in the central area between all groups 30 minutes after the 1^st^ i.p. injection in the open field study (see Figures 1 and 2).

### 3.3. Risperidone + MK-801

The results showed that the dose 0.1 mg/kg of risperidone could totally inhibit the MK-801-induced hyperlocomotion compared to that of the vehicle + MK-801 group (*F* = 17.18, *p* < 0.001) (see Figure 1).

For further imaging analysis for risperidone groups, see Figure (a) of Supplements 3–6.

### 3.4. Valproic Acid + MK-801

Valproic acid (200 mg/kg) attenuated the MK-801-induced hyperlocomotion. The travelled behavioral patterns showed the persistent partial suppression on the MK-801 effect at various corresponding time points (see Figure 2). However, the overall level of suppression is less significant comparing with that of risperidone.

For further imaging analysis of the valproic acid group, see Figure (b) of Supplements 3–6.

### 3.5. Differences of Risperidone/Valproic Acid on MK-801 Effects

There are the significant differences in the total travelled distance between vehicle + MK-801 (255 ± 51 meters) and risperidone + MK-801 (43 ± 3 meters) (*p* < 0.001); while valproic acid showed partial suppression on MK-801-induced hyperlocomotion, the total travelled distances were 257 ± 39 and 194 ± 12 meters in vehicle + MK-801 and valproic acid + MK-801 (*p* = 0.2706), respectively (see Table 1).

The significant increase of the central travelled distance, speed, and duration was noted following the MK-801 injection (*p* < 0.001). There were no significant differences between the vehicle + vehicle group, risperidone + vehicle group, and risperidone + MK-801 groups in the total travelled distance and speed, central travelled distance and speed, duration in central area, and frequency of entering the central area (see Figure 3(c)). The differences in the central travelled distance, central travelled speed, and frequency of entering the central area were seen between the MK-801-injected groups and those that did not receive MK-801, in which the groups receiving MK-801 (vehicle + MK-801 and valproic acid + MK-801) were significantly higher (*F* = 15.45, *p* < 0.01 central travelled distance; *F* = 17.97, *p* < 0.001 in frequency of entering the central area) (see Figure 3(b)). Significantly higher duration in the central area was found between the valproic acid + MK-801 group and those without MK-801 (*F* = 8.51, *p* < 0.01) (see Figure 3(d)).

### 3.6. Gaiting Analysis

**Figure 4:**
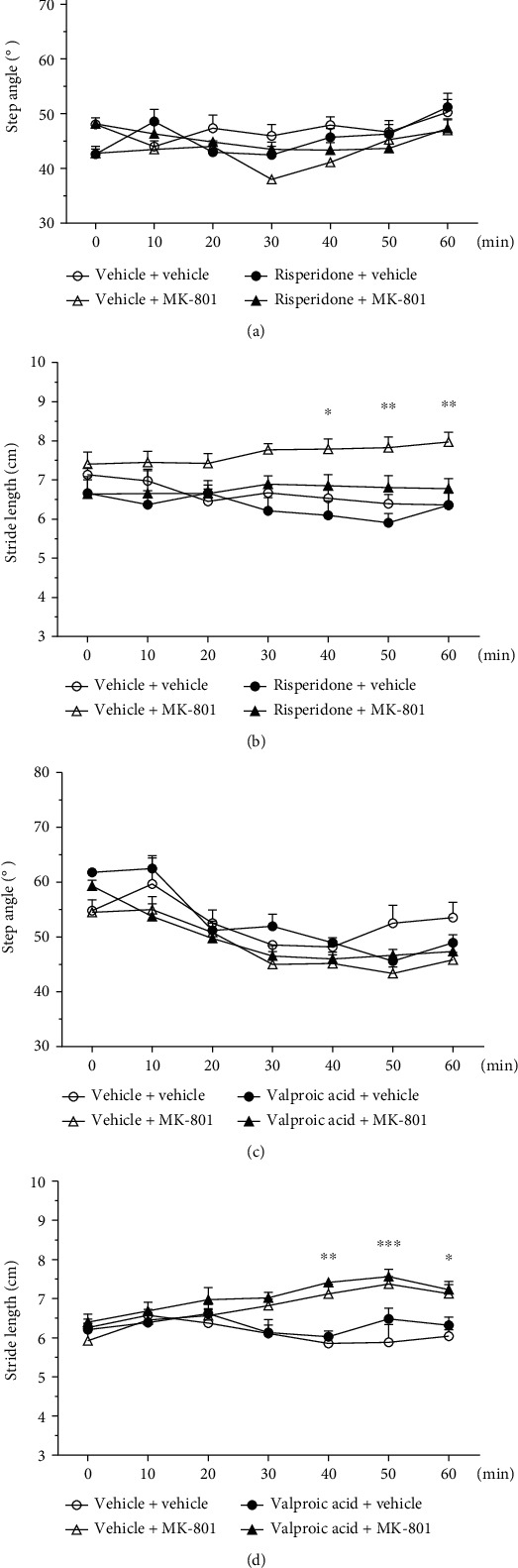
Behavioral differences of gaiting, step angle (a, c), and stride length (b, d) followed by MK-801 injection for mice treated with risperidone/valproic acid. Statistically significant differences between groups: ^∗^*p* < 0.05, ^∗∗^*p* < 0.01, and ^∗∗∗^*p* < 0.001 vs. vehicle + vehicle. Vertical bars represent SEMs.

In the gaiting test, the stride length was significantly increased after MK-801 injection (*p* < 0.05 40 minutes after MK-801 injection and *p* < 0.01 50 and 60 minutes after MK-801 injection) and there is no difference in other parameters regarding the step angle and stance width (see Figure 4(a)). The valproic acid or risperidone alone did not affect the gaiting behaviors including the analyses of the step angle, stride length, and stand width.

The increased stride length of mice after MK-801 injection could be reduced by prior the administration of dose 0.1 mg/kg of risperidone, and there were no significant differences between risperidone + MK-801, risperidone + vehicle, and vehicle + vehicle groups regarding stride length (see Figure 4(b)). The gaiting test showed that valproic acid could not alter the gaiting of MK-801-injected subjects. As seen in the risperidone groups, there is also an increase of stride length after MK-801 injection. However, there were no differences in the step angle and stance width between all groups receiving MK-801 (see Figure 4(c)) and both the vehicle + MK-801 group and the valproic acid + MK-801 group showed a significant increase in stride length comparing with the control group (*p* < 0.01 40 minutes after MK-801 injection, *p* < 0.001 50 minutes after MK-801 injection, and *p* < 0.05 60 minutes after MK-801 injection) (see Figure 4(d)).

## 4. Discussion

In this study, the homogeneity of the mice strain, age, sex, and environmental factors, such as illumination, temperature, and humidity is under well control, providing a good platform to assay the neuropharmacological effects of valproic acid and risperidone following MK-801, a specifically NMDA receptor antagonist. The principal findings of this study have shown the following: (1) MK-801 persistently produces hyperlocomotion, (2) locomotor behaviors are unaffected by risperidone and valproic acid injection alone, (3) risperidone markedly suppresses the MK-801-induced hyperlocomotion, and (4) valproic acid partially inhibits the MK-801 pharmacological effects. To our knowledge, this is the first report to explore the pharmacological differences of risperidone and valproic acid in a same valid module, as comparing MK-801 modulation on the blockade of glutamatergic neurotransmission.

### 4.1. Drug Effects on Locomotor Activities

In this study, 0.2 mg/kg of MK-801 was employed because <0.1 mg/kg of dose may be insufficient enough to induce hyperlocomotor activity and > 0.5 mg/kg of dose may induce ataxia in male mice which could intervene with this study [32, 33]. Doses of 0.1 mg/kg of risperidone and 200 mg/kg of valproic acid were employed based on previous related studies [34–38]. During the open field test, the MK-801-injected subjects displayed hyperlocomotion, increased frequency of entering the central area, increased total and central speed, and increased central distance, indicating the psychotomimetic effect of MK-801 resulting in overall agitation and disorganized behaviors in test subjects. The study result is consistent with the open field test of the schizophrenia glutamate model [6, 39], proposing the excitatory schizophrenia symptoms which resulted from NMDA inhibition. In this study, the hyperlocomotion could be completely suppressed by prior risperidone injection (0.1 mg/kg), implying that antagonizing of dopamine receptors, especially dopamine receptor D2, and serotonin (5-hydroxytryptamine; 5-HT) receptors, especially the 5-HT_2A_ receptor, which are main targets of atypical antipsychotics, plays a major role in inhibiting the excitation and agitated behaviors. The previous study demonstrated that antagonizing of D1 and D2 receptors, as well as 5-HT_2A_, is necessary for the suppression of MK-801-induced hyperlocomotion [40]. Similarly, there are evidences suggesting that the psychotomimetic effects illustrated in the hyperlocomotor activity of MK-801 along with other NMDA antagonists are believed to result from the increase of the dopamine level in the prefrontal cortex [5, 41]. It is noteworthy that the agitated and excited behaviors seen in various neuropsychiatric diagnoses may be antecedent from the dopamine hyperactivation and there are also evidences that such increase in dopamine neuron firing is regulated by NMDA-mediated glutamatergic neurons [5, 41]. The positive psychotomimetic effects of ketamine and PCP are believed to be derived from the inhibition of the NMDA receptor on the *γ*-aminobutyric acid (GABA) neuron, resulting in hyperdopaminergic transmission [42, 43]. Being a structural analog of PCP and ketamine [44], MK-801 acts on the central glutamate NMDA receptor, inhibits the glutamatergic transmission, may lead to decreased GABAergic neuron firing, and disinhibits dopamine neurotransmission [45].

### 4.2. Drug Effects on the Gaiting Test

Other than hyperlocomotor activity, the acute MK-801 administration can also result in a specific motor pattern of ataxia and stereotypy in injected mice which may interfere with the open field performances [15], and in this study, the gaiting test was performed to evaluate the behavioral quality of the mice after receiving testing drugs. The significant differences in stride length of the vehicle + MK-801 group vs. vehicle + vehicle group indicated the behavioral change in MK-801 injection, as the tested mice tended to take longer steps after injection. The MK-801 induced longer stride lengths and was rescued by risperidone treatment, as shown in Figures 3 and 4 of Supplement 7. The result is compatible to the findings in the open field test as reflected in the increased total travelled distance and travelling speed [31]. It may indicate that the increased stride length after MK-801 injection is a phenomenon of excitation/agitation rather than drug adverse effects such as ataxia or stereotypy. The MK-801 + valproic acid group showed no such reduction in stride length, suggesting an inferior ability of valproic acid comparing to risperidone in glutamatergic suppression-induced excitation/agitation, as shown in Figures 3 and 4 of Supplement 8 (see Figure 4(d)). In addition, no differences were observed between the risperidone + vehicle group and vehicle + vehicle group regarding all gaiting parameters including the step angle, stride length, and stance width, suggesting that the antipsychotic suppression of the hyperlocomotion seen in the open field test is not a result of the drug effect over the subject's movement quality, but a suppression of hyperlocomotion.

### 4.3. Interplay of Neurobehaviors and Pharmacodynamic Effects between Drugs

In this study, the valproic acid- (200 mg/kg) injected subjects showed partial suppression over the hyperlocomotion induced by MK-801, implying an adjuvant effect over the excitation and agitation behaviors of acute neuropsychiatric symptoms. It is not uncommon that valproic acid is utilized in treating patients with agitation/excitation symptoms; while valproic acid monotherapy for manic episode in bipolar affective disorder patients is frequently used with good clinical evidence, it is rarely used as monotherapy in schizophrenia or substance intoxication/withdrawal patients. It may raise questions that whether the agitated and excitation behaviors presented in each disorder have a common mechanism and could be all managed by valproic acid. There have been preliminary investigations of valproic acid use in schizophrenia patients [27, 46], especially in violent and aggressive patients, but with mixed results. Up to this date, valproic acid monotherapy is not approved for the treatment of schizophrenia; in most articles and treatment guidelines, valproic acid remains as augmentation treatment strategy but showed being ineffective in the treatment of schizophrenia core symptoms such as hallucinations or delusions [21–24, 47]. Since the first marketing in the 1960's, valproic acid is initially used for the treatment of epilepsy and later discovered to be effective in various excitability disturbances in both central and peripheral nervous system disorders, including neuropathic pain, migraine, bipolar disorder, essential tremor, and even schizophrenia [48–50].

Valproic acid is suggested to possess multiple mechanisms of action, the major actions including the potentiation of GABAergic neuron, the blockade of voltage-dependent sodium channels, the blockade of T-type calcium channels, and the blockade and modulation of glutamatergic excitation neurons [48–50]. As mentioned above, these mechanisms of action could explain its overall inhibitory effects over neural systems of valproic acid and why it is favored in the use to control symptoms believed to cause neuron excitation, such as epilepsy. However, as demonstrated in this study, valproic acid only possesses modest effect over MK-801 or NMDA-mediated glutamate excitation behaviors comparing with risperidone. Besides the total travelled distance, the valproic acid + MK-801 group showed no difference versus the vehicle + MK-801 group in the central travelled distance, central speed, and frequency of the central entry in the open field test, in contrary to the risperidone + MK-801 group, which revealed suppression in the above parameters comparing with the vehicle + MK-801 group. In addition to open field test findings, the gaiting test also confirmed that valproic acid could not reverse the abnormal behavioral pattern induced by MK-801. This study result is compatible with the current treatment strategy for schizophrenia. Risperidone revealed a more dominant effect than valproic acid to rescue MK-801 induced hyperlocomotion, which is consistent with the clinical therapeutic effect of risperidone on acute psychosis. It may imply that the agitated/impulsive behaviors shown in schizophrenia indeed have a distinct disease entity other than what is seen in bipolar affective disorder but has some degree of overlapping which could be partially managed by adjunctive of valproic acid but could not be treated by valproic acid alone [25].

The study indicated that the partial suppression effect of valproic acid on MK-801-induced hyperlocomotion may have some similarities in these psychiatric diagnoses regarding the drug effect and neurological mechanism. Our results demonstrate MK-801 augmentation of locomotor activity via NMDA receptor blockade [6]. It might imply that the compound could attenuate the GABA function and caused the overactivity of dopaminergic neurotransmission. The dominant inhibitory effect of risperidone on MK-801 is via the inhibition of dopamine transmission. The neurobehavioral alterations of MK-801 and risperidone would raise the potential interactions of pharmacological properties between glutamate and dopamine neurotransmissions. Apart from the glutamate-dopamine crosstalk, valproic acid has been used as a psychotropic agent through balancing the GABA and glutamate neurofunction. It may provide neuropharmacological evidence for valproic acid in adjuvant to risperidone to reduce psychosis and also encourages further examining of the nature of the agitation/excitement phenotype of psychiatric patients and to consider a different treatment approach strategy.

The study adopted the open field to evaluate locomotor activity and gaiting to assess the behavioral quality, in which the drug suppression effect of MK-801 induced hyperlocomotion mimicking the treatment response of acute psychosis in the animal model. Apart from the acute excitation, the current study module could not totally encompass the diverse symptom domain in acute psychotic subjects clinically; therefore, further study is required to explore the other symptom domains such as fearfulness, anxiety, cognitive, and negative symptoms. There are other animal models for psychotic symptoms, including prepulse inhibition (PPI), latent inhibition, social interaction and recognition, forced swim test, and other models for cognitive symptoms [5]. It can be insightful to integrate other animal models in this study module. Besides the study management, it will be an interesting issue to compare the drug reactions in other mouse strains with different genetic backgrounds. In addition, this study mostly adopted adult mice for test subjects and evidence indicated that age differences of mice test subjects will be a confounding factor to the drug sensitivity and susceptibility, especially in adolescence mice [51, 52]. Further studies also aim to integrate more sample size and comprehensive animal neurobehavioral models to clarify the valproic acid on the treatment effect of psychosis. Finally, this study examined the effect of antipsychotic and valproic acid over MK-801-induced hyperlocomotion alone but the augmentation treatment strategy with antipsychotics plus valproic acid for the acute excitation subjects was not tested and is left for future investigation, especially the interactions of dopamine, GABA, and glutamate neurotransmission.

## 5. Conclusion

The more dominant effect of risperidone to rescue MK-801 induced hyperlocomotion compared with valproic acid. The partial suppression of valproic acid may imply the psychopharmacological evidence as adjuvant effect to treat psychotic patients through tuning glutamatergic neurotransmission. It would be an important issue to deeply investigate our current findings through integrating the neurophysiology, neurobiology, and neuroimaging studies furthermore.

## Figures and Tables

**Table 1:** Comparisons of risperidone and valproic acid effects on MK-801-induced hyperlocomotion.

	Risperidone dose		Valproic acid dose	
MK-801 dose (mg/kg)	0 mg/kg	0.1 mg/kg	0 mg/kg	200 mg/kg
0	67.2 ± 10.3	25.2 ± 6.0	58.0 ± 14.4	40.4 ± 5.6
0.2	255 ± 51.2^∗∗^	42.5 ± 4.3^†††^	257 ± 39.2^∗∗∗^	194 ± 12.3^∗∗,§§^

Values are means ± SEM. Data represent the total travelled distance (meter) in 60 minutes following MK-801 injection. Differences among the study group were evaluated by multiple analysis of variance and post hoc analysis. Subject numbers were 9–11 in each group. ^∗∗^*p* < 0.01 and ^∗∗∗^*p* < 0.001 vs. the 0 mg/kg MK-801 group; ^†††^*p* < 0.01 vs. the 0 mg/kg risperidone group; ^§§^*p* < 0.01 vs. the 200 mg/kg valproic acid + 0 mg/kg MK-801 group.

## Data Availability

The data utilized to support the findings are available from the corresponding authors upon request.

## Supplementary Materials

Supplementary Materials The study procedure was shown in Supplement Figure 1, and the mouse movement tracking was shown in Supplement Figure 2. The parameters of the study measurement including central frequencies, central duration percentages, central travelled speed, and overall travelled speed were shown in Supplement Figures 3–6. Supplements Figures 7 and8 demonstrated the behavioral gaiting following risperidone and valproic acid treatment, respectively.
