# Supplementary material for: Neurobehavioral Differences of Valproate and Risperidone on MK-801 Inducing Acute Hyperlocomotion in Mice
Source: Behav Neurol. 2022 Feb 23;2022:1048463. doi: 10.1155/2022/1048463 (PMC8890888; doi:10.1155/2022/1048463)
Supplement: Supplementary Materials — The study procedure was shown in Supplement Figure 1, and the mouse movement tracking was shown in Supplement Figure 2. The parameters of the study measurement including central frequencies, central duration percentages, central travelled speed, and overall travelled speed were shown in Supplement Figures 3–6. Supplements Figures 7 and8 demonstrated the behavioral gaiting following risperidone and valproic acid treatment, respectively. [file 1048463.f1.pdf]

Supplement 1. Experimental design

**Subjects**	**Methods**
Strain: C57BL/6J<br>Age group: Adult mice<br>N = 9-11 each group	Measurement<br>– Open field (Locomotion)<br>– Video tracking system<br>– Gaiting test

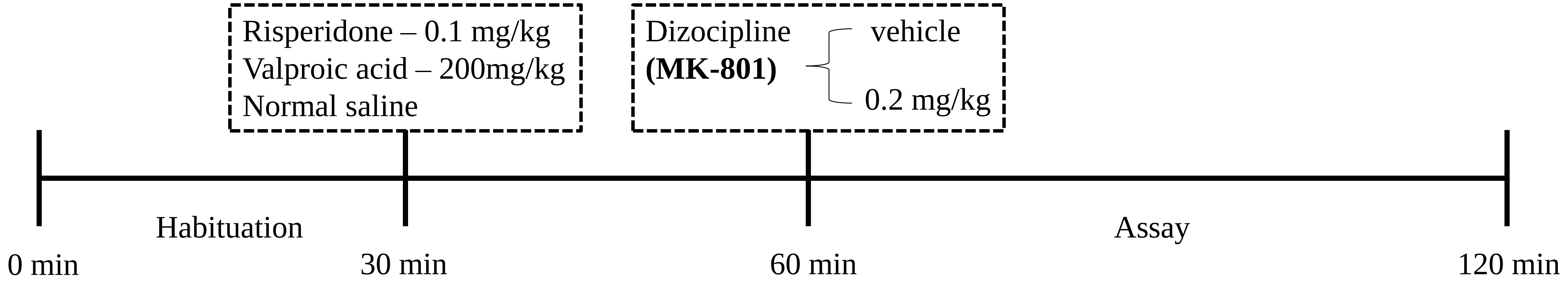

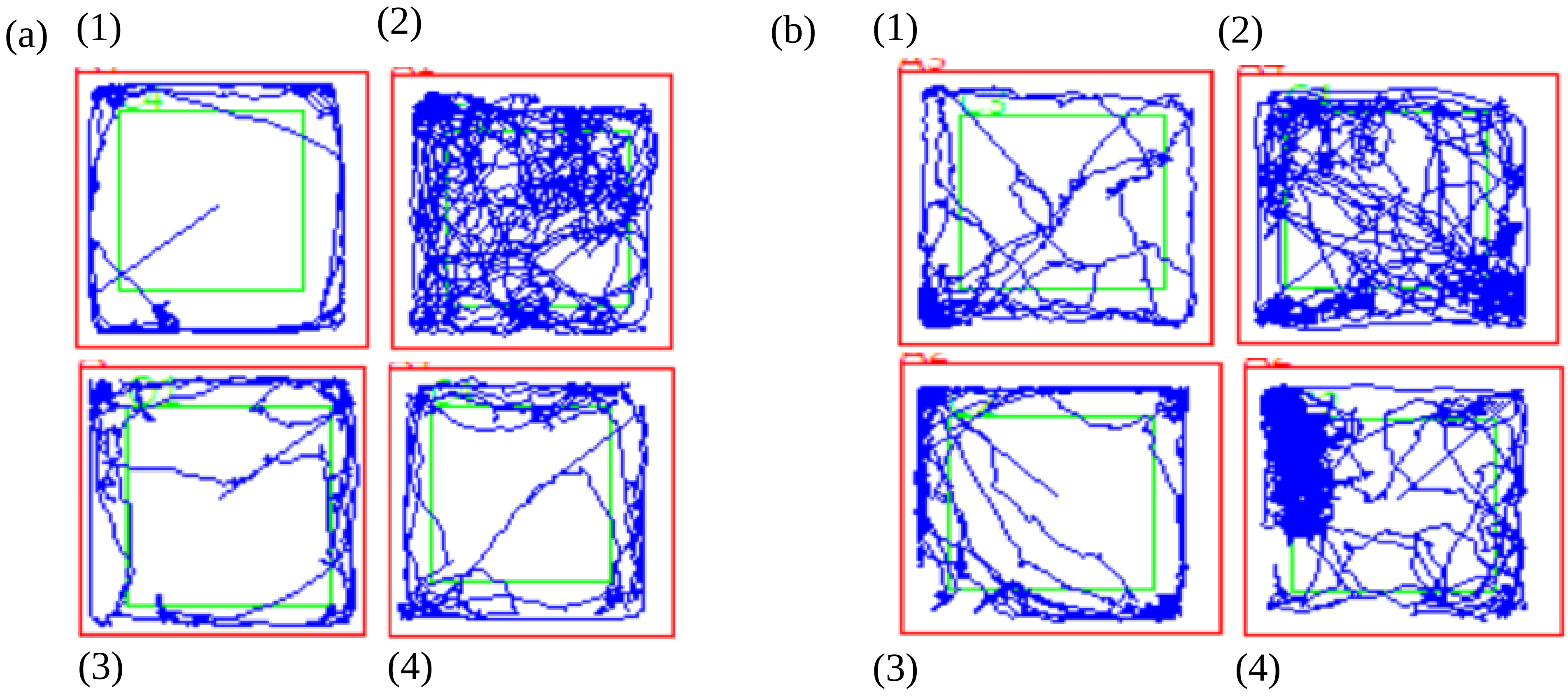

Supplement 2. The lines illustrate the total travelled distance in open field test of (a) risperidone + MK-801 groups 15 minutes following MK-801 i.p. injection regarding the (1) vehicle + vehicle, (2) vehicle + MK-801, (3) risperidone + vehicle, and (4) risperidone + MK-801 groups; and the total travelled distance of (b) valproic acid + MK-801 groups 15 minutes following MK-801 i.p. injection regarding the (1) vehicle + vehicle, (2) vehicle + MK-801, (3) valproic acid + vehicle, and (4) valproic acid + MK-801 groups.

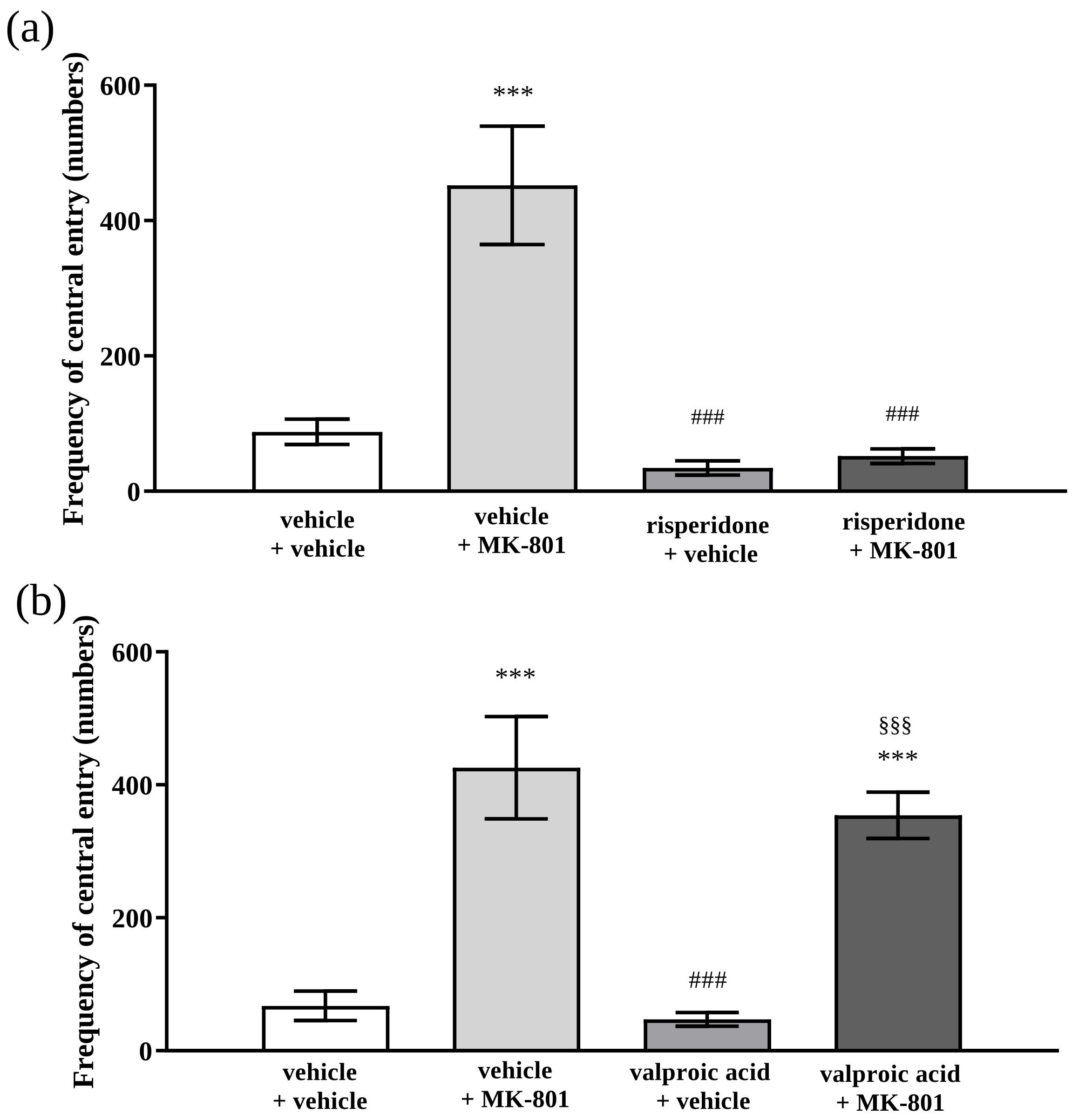

Supplement 3. Frequency of central entry (numbers) in the open field tests within 60-120 minutes regarding (a) risperidone groups and (b) valproic acid groups. Statistically significant difference between groups: * $p < 0.05$, ** $p < 0.01$, *** $p < 0.001$ vs. vehicle + vehicle; # $p < 0.05$, ## $p < 0.01$, ### $p < 0.001$ comparing to vehicle + MK-801; § $p < 0.05$, §§ $p < 0.01$, §§§ $p < 0.001$ vs. valproic acid + vehicle.

(a)

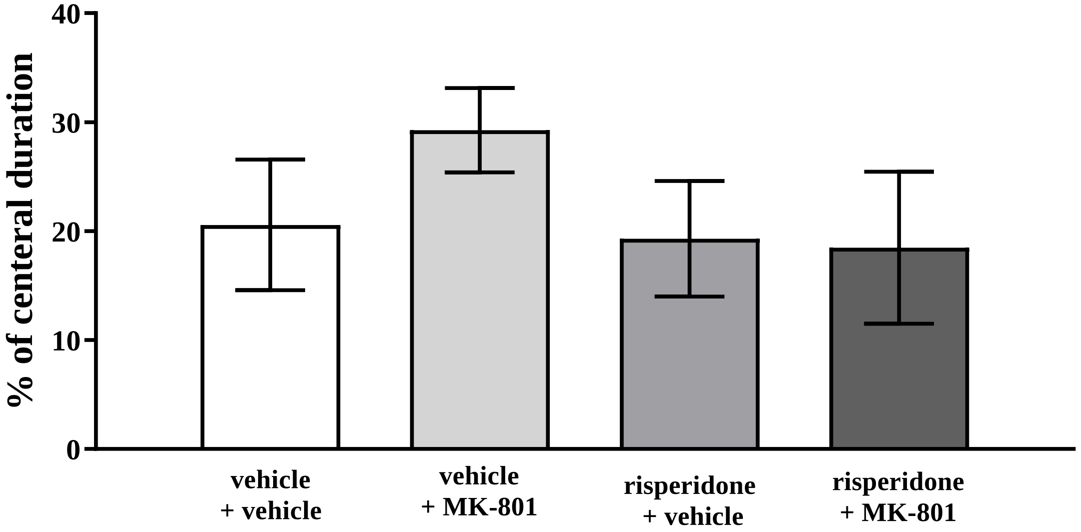

(b)

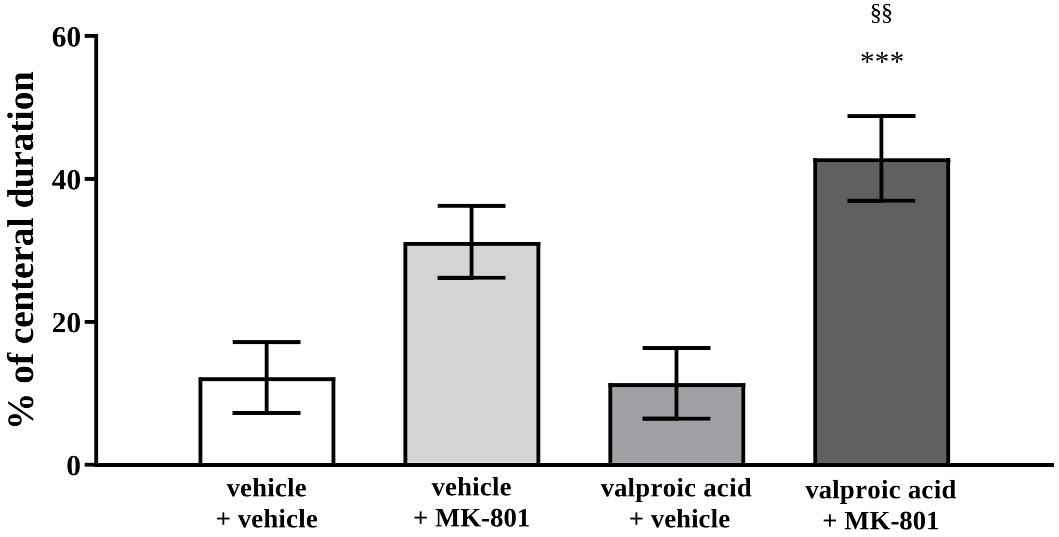

Supplement 4. Percentage of central duration comparing to total duration in the open field tests within 60-120 minutes regarding (a) risperidone groups and (b) valproic acid groups. Statistically significant difference between groups: * $p < 0.05$, ** $p < 0.01$, *** $p < 0.001$ vs. vehicle + vehicle; § $p < 0.05$, §§ $p < 0.01$, §§§ $p < 0.001$ vs. valproic acid + vehicle.

(a)

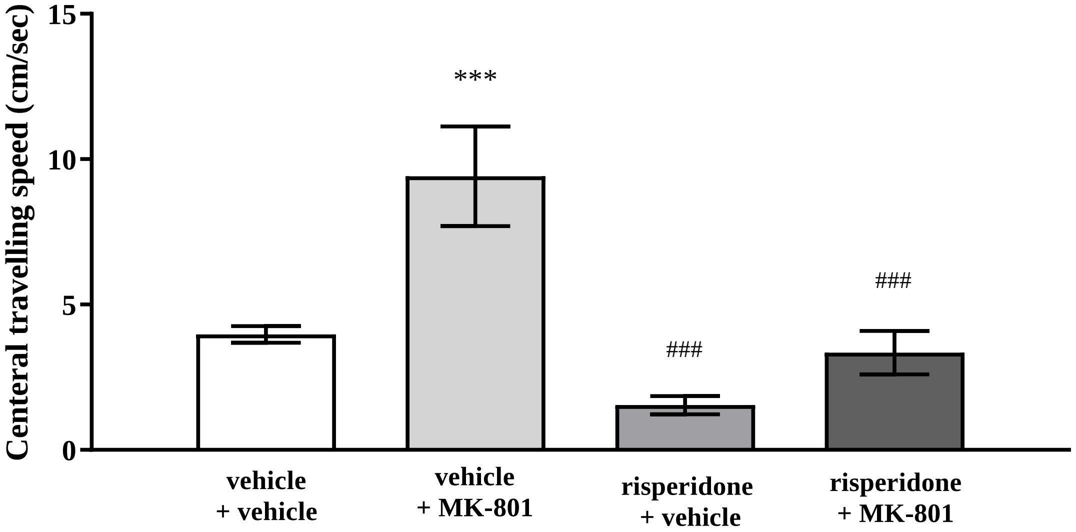

(b)

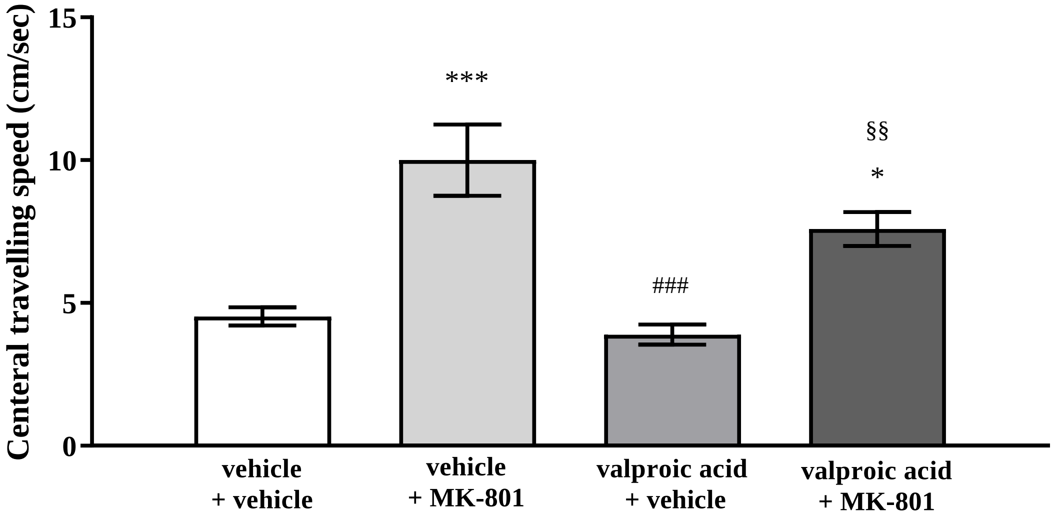

Supplement 5. Central area travelled speed in the open field tests within 60-120 minutes regarding (a) risperidone groups and (b) valproic acid groups. Statistically significant difference between groups: * $p < 0.05$, ** $p < 0.01$, *** $p < 0.001$ vs. vehicle + vehicle; # $p < 0.05$, ## $p < 0.01$, ### $p < 0.001$ comparing to vehicle + MK-801; § $p < 0.05$, §§ $p < 0.01$, §§§ $p < 0.001$ vs. valproic acid + vehicle.

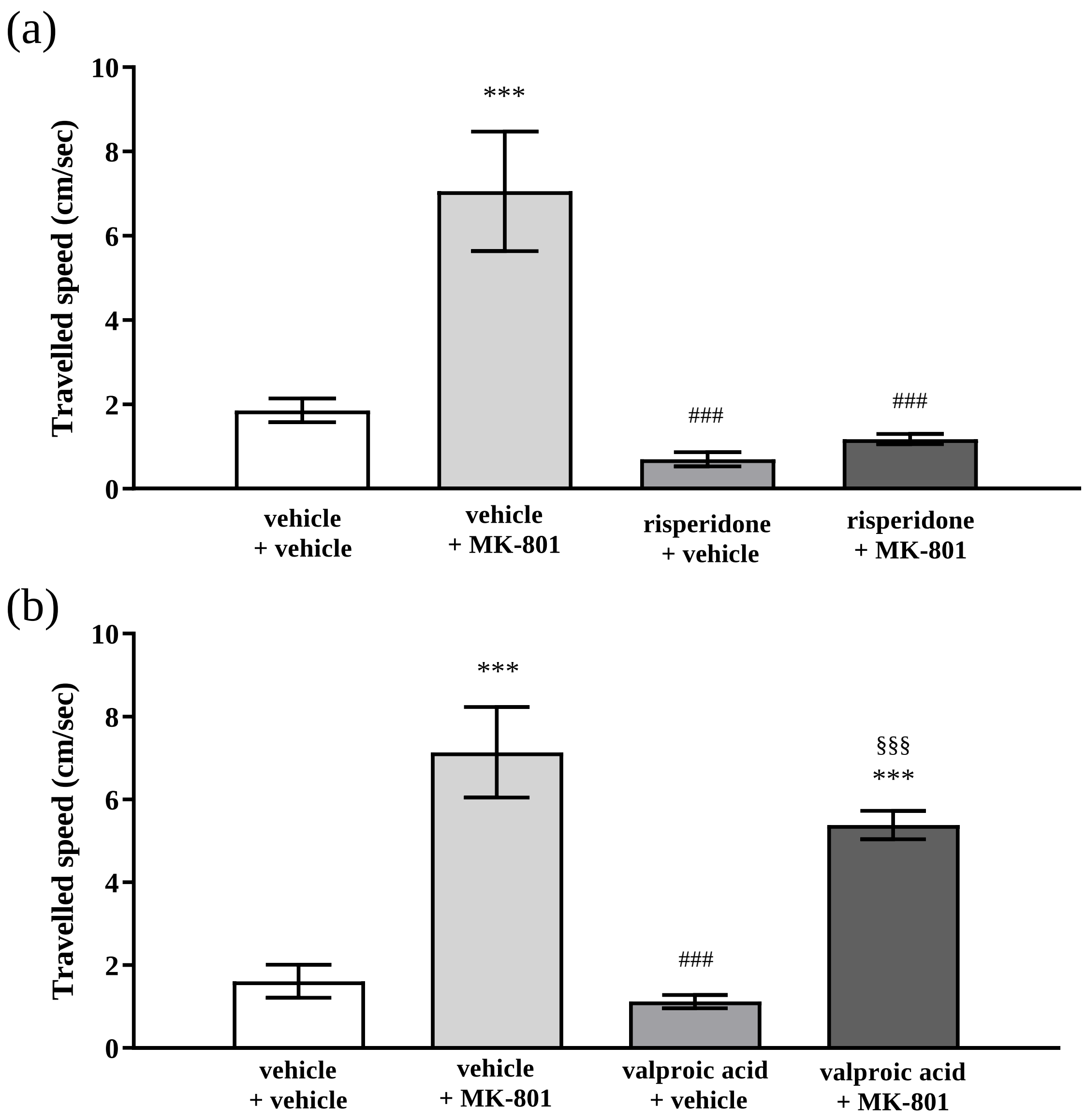

Supplement 6. Travelled speed in the open field tests within 60-120 minutes regarding (a) risperidone groups and (b) valproic acid groups. Statistically significant difference between groups: * $p < 0.05$, ** $p < 0.01$, *** $p < 0.001$ vs. vehicle + vehicle; # $p < 0.05$, ## $p < 0.01$, ### $p < 0.001$ comparing to vehicle + MK-801; § $p < 0.05$, §§ $p < 0.01$, §§§ $p < 0.001$ vs. valproic acid + vehicle.

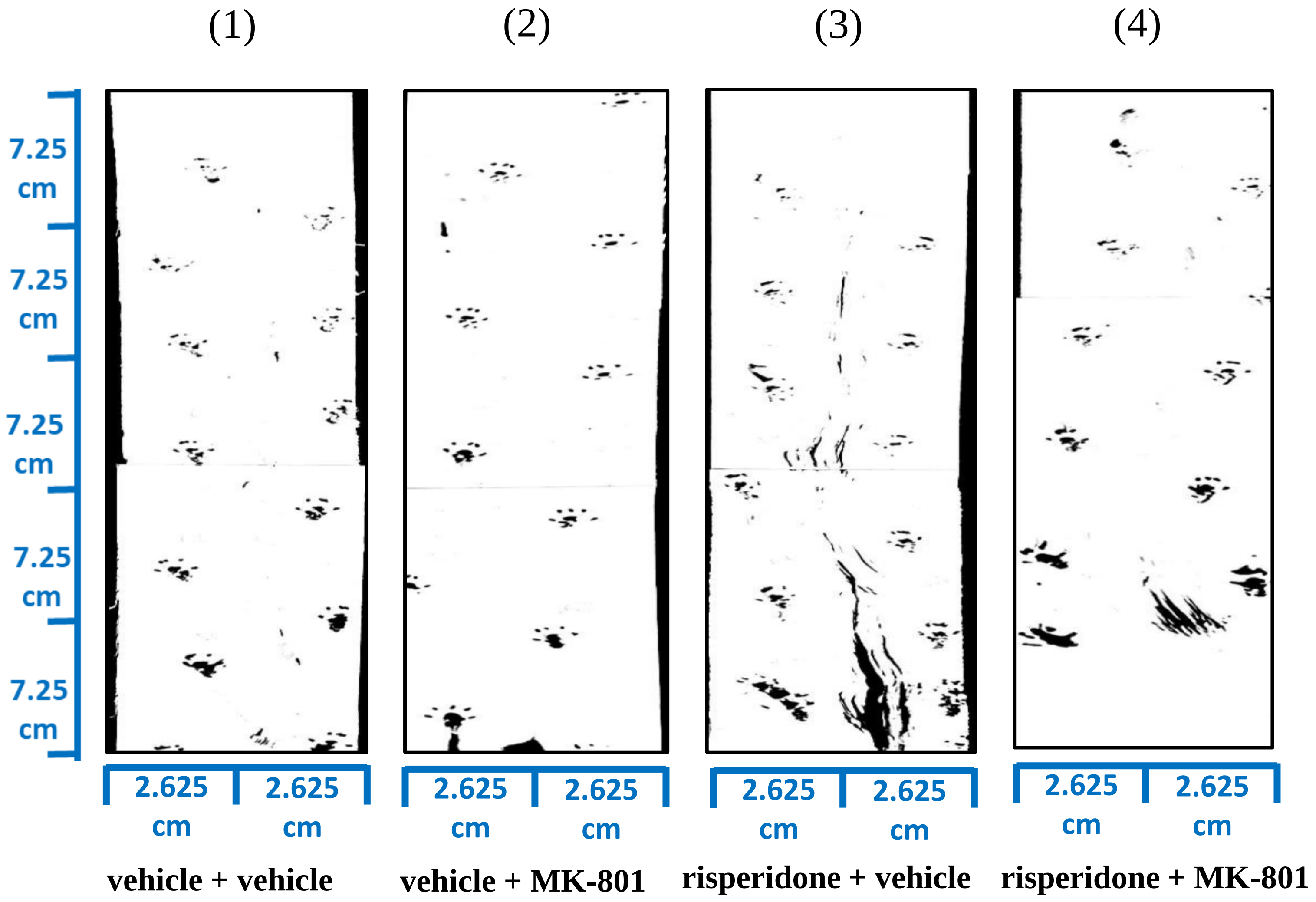

Supplement 7. Gaiting records of the risperidone groups: (1) vehicle + vehicle group (2) vehicle + MK-801 group (3) risperidone + vehicle group (4) risperidone + MK-801 group.

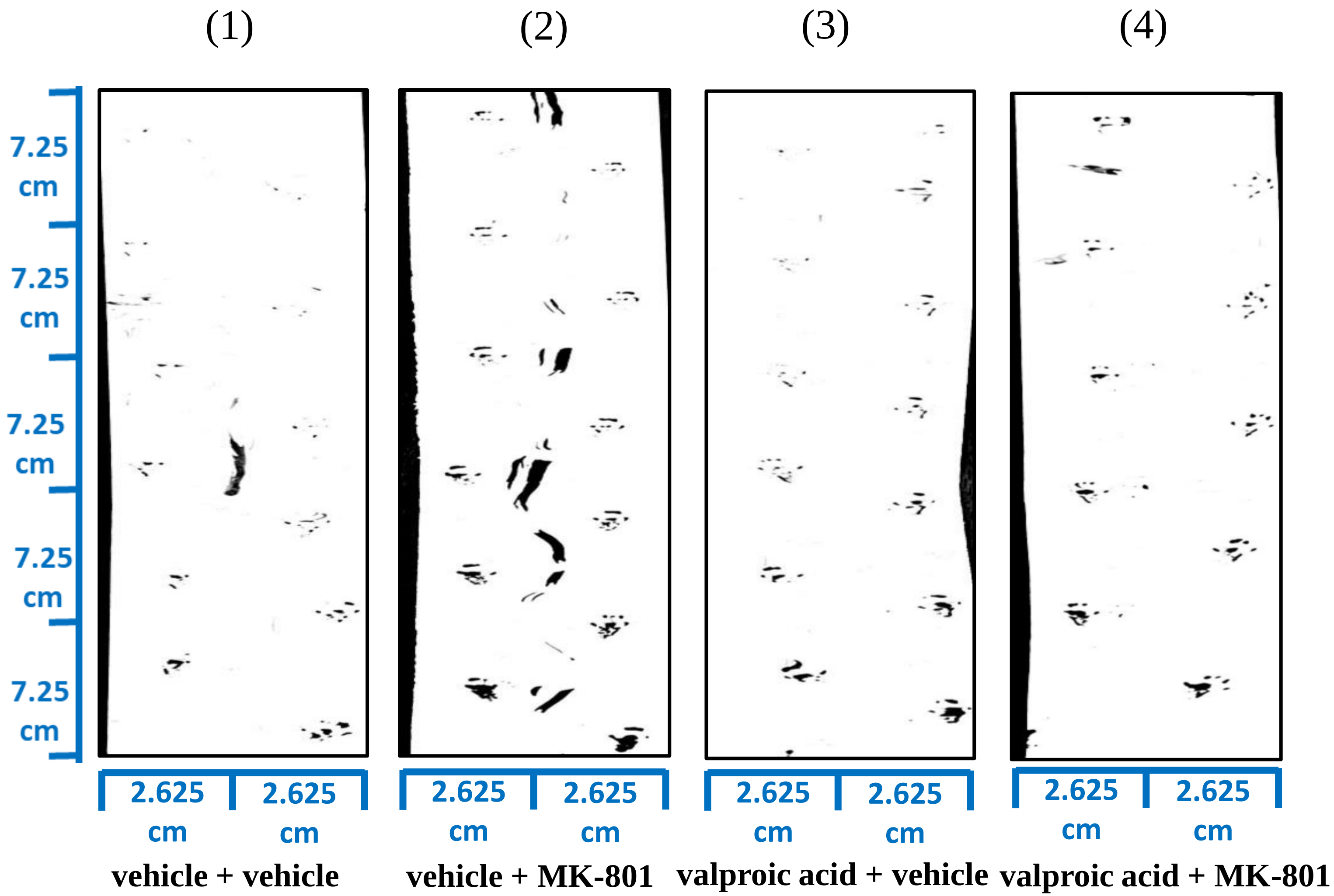

Supplement 8. Gaiting records of the valproic acid groups: (1) vehicle + vehicle group (2) vehicle + MK-801 group (3) valproic acid + vehicle group (4) valproic acid + MK-801 group.
